# Implementation of a peer support worker in a forensic hospital in germany

**DOI:** 10.1192/j.eurpsy.2021.88

**Published:** 2021-08-13

**Authors:** P. Walde, C. Benz, B. Völlm

**Affiliations:** 1 Klinik Für Forensische Psychiatrie, Universitätsmedizin Rostock, Rostock, Germany; 2 Forensic Psychiatry, University of Rostock, Rostock, Germany

**Keywords:** forensic mental health, peer support work, recovery

## Abstract

Experienced Involvement (also called Peer Support Work, PSW) has existed in mental health care in Germany since 2005 though its implementation lags behind, compared to other countries. Due to the unique challenges of forensic-psychiatric settings, implementation of PSW in these settings is even less developed. We prepared the implementation of a peer support worker in our forensic hospital for addicted offenders in Germany in several steps: A survey amongst the 75 forensic hospitals in Germany was conducted to evaluate the prevalence of PSW in these settings. Individual interviews were conducted with directors and peer support workers of forensic clinics nation-wide to investigate their facilities’ experiences with PSW. Focus groups with several occupational groups of the clinic in Rostock addressed staffs opinions, expectations and reservations regarding peer support work. These were recorded and transcribed for thematic analysis. **Results:** revealed that the majority of forensic hospitals (83.6%) has no experience with peer support work. Interviews with external clinic directors revealed similar concerns and expectations among the employees as our focus groups did. Staff at the clinics expected the peer support worker to offer useful experiences and new perspectives. Concerns occurred about stability of health condition of the peer support worker, trust issues because of former criminal behavior and attitudes towards psychiatric treatment that might interfere with professional treatment negatively. Furthermore the clinic directors stressed the importance of a well prepared implementation and a good “fit” of the peer support workers background to the patients (e.g. regarding diagnosis).

**Disclosure:**

No significant relationships.

